# Glycosylated, Lipid-Binding, CDR-Like Domains of SARS-CoV-2 ORF8 Indicate Unique Sites of Immune Regulation

**DOI:** 10.1128/spectrum.01234-23

**Published:** 2023-06-15

**Authors:** Fang Wu, Xin Chen, Yanhong Ma, Yuzhe Wu, Rui Li, Yuanwei Huang, Rong Zhang, Yaoqi Zhou, Jian Zhan, Shuwen Liu, Wei Xu

**Affiliations:** a Guangdong Provincial Key Laboratory of New Drug Screening, School of Pharmaceutical Sciences, Southern Medical University, Guangzhou, China; b Institute for Systems and Physical Biology, Shenzhen Bay Laboratory, Shenzhen, Guangdong, China; c Key Laboratory of Medical Molecular Virology (MOE/NHC/CAMS), School of Basic Medical Sciences, Shanghai Medical College, Biosafety Level 3 Laboratory, Fudan University, Shanghai, China; Karolinska Institutet

**Keywords:** SARS-CoV-2, ORF8, glycosylation, lipid-binding protein, immune-mediated protein

## Abstract

The outbreak of the novel coronavirus SARS-CoV-2 has posed a significant threat to human health and the global economy since the end of 2019. Unfortunately, due to the virus’s rapid evolution, preventingand controlling the epidemic remains challenging. The ORF8 protein is a unique accessory protein in SARS-CoV-2 that plays a crucial role in immune regulation, but its molecular details are still largely unknown. In this study, we successfully expressed SARS-CoV-2 ORF8 in mammalian cells and determined its structure using X-ray crystallography at a resolution of 2.3 Å. Our findings reveal several novel features of ORF8. We found that four pairs of disulfide bonds and glycosylation at residue N78 are essential for stabilizing ORF8’s protein structure. Additionally, we identified a lipid-binding pocket and three functional loops that tend to form CDR-like domains that may interact with immune-related proteins to regulate the host immune system. On cellular experiments also demonstrated that glycosylation at N78 regulats of ORF8’s ability to bind to monocytes cells. These novel features of ORF8 provide structural insights to into its immune-related function and may serve as new targets for developing ORF8-mediated immune regulation inhibitors.

**IMPORTANCE** COVID-19, caused by the novel coronavirus SARS-CoV-2 virus, has triggered a global outbreak. The virus’s continuous mutation increases its infectivity and may be directly related to the immune escape response of viral proteins. In this study, we used X-ray crystallography to determine the structure of SARS-CoV-2 ORF8 protein, a unique accessory protein expressed in mammalian cells, at a resolution of 2.3 Å. Our novel structure reveals important structure details that shed light on ORF8’s involvement in immune regulation, including conservation disulfide bonds, a glycosylation site at N78, a lipid-binding pocket, and three functional loops that tend to form CDR-like domains that may interact with immune-related proteins to modulate the host immune system. We also conducted preliminary validation experiments on immune cells. These new insights into ORF8’s structure and function provide potential targets for developing inhibitors to block the ORF8-mediated immune regulation between viral protein and host, ultimately contributing to the development of novel therapeutics for COVID-19.

## INTRODUCTION

Since the end of 2019, the global pandemic of COVID-19, caused by severe acute respiratory syndrome coronavirus 2 (SARS-CoV-2), has posed a significant threat to public health and the global economy worldwide. Globally, there have been over 661 million confirmed cases, resulting in 6.7 million deaths (WHO, 2022, https://covid19.who.int/). COVID-19 is characterized by a prolonged incubation period, extended clinical infection, and long time for the virus to clear in patients, suggesting that SARS-CoV-2 is a novel, highly pathogenic betacoronavirus that significantly divergents from other human-infecting coronaviruses, such as SARS-CoV and MERS-CoV (1). Phylogenetic analysis has shown that SARS-CoV-2 is closely related to bat-SL-CoVZC45 and bat-SL-CoVZXC21, but genetically distinct from SARS-CoV (2). The prevalence of asymptomatic infections has presented a significant challenge in controlling epidemic, leading us to reconsider the structural changes of important viral proteins and their roles in the difference between SARS-CoV-2 and other coronaviruses.

The genome of SARS-CoV-2 encodes 29 proteins that can be categorized into structural proteins, non-structural proteins, and accessory proteins. Four structural proteins, namely, the spike (S), envelope (E), membrane (M), and nucleocapsid (N) protein, play crucial roles in genome packaging, virion integrity and the virus’s entry into the host cells (3). The 16 nonstructural proteins are mainly involved in transcription and replication of the viral genome, while the nine accessory proteins located at the downstream of the genome are thought to aid viral immune evasion and modulate the defense mechanisms against the host, including open reading frames (ORFs) 3a, 3b, 6, 7a, 7b, 8, 9a, 9b, and 10 (4). A comparison of the complete viral genome between SARS-CoV-2 and SARS-CoV revealed that, despite an 80% similarity in their genetic nucleotides, the similarity of ORF8 amino acid residues between these two viruses is less than 20% (5).

The specific and unique sequence of SARS-CoV-2 ORF8, along with the strongest specific antibody responses, has been used as a clinical diagnosis marker for COVID-19 (6). This may contribute to the differences in clinical symptoms SARS-CoV-2 and SARS-CoV. ORF8 is a unique accessory gene found in the SARS-CoV-2 genome, and its specificity distinguishes it from other coronaviruses, making it an accurate clinical diagnosis marker for COVID-19 (6). ORF8 has been found to stimulate the strongest specific antibody responses, contributing to the accuracy of clinical diagnosis in serological detection at both early and late stages of COVID-19 (7). The evolved immunoglobulin-like (Ig-like) viral proteins play a role in mediating interaction with major histocompatibility complex class I (MHC-I), rendering the virus undetectable in infected cells to evade the host immune system and suppress the host immune response (8). ORF7a, a closely related accessory protein in structure to ORF8, is also a member of the Ig-like proteins, confirming that the Ig-like fold is a key domain that interferes with the host immune system (9). As a viral Ig-like domain protein, ORF8 plays a critical role in mediating host immune responses. The rapid evolution of ORF8 directly affects its interference with the immune system (10). In other words, the structural characteristics of various coronavirus ORF8 proteins determine their biological functions (11).

Protein ORF8 is one of the most evolving betacoronavirus proteins and may have contributed to immune evasion of the virus, leading to greater infectivity and confirmed cases of SARS-CoV-2 (12). Three potential immunomolecular targets of SARS-CoV-2 ORF8 have been reported (13–15). Patients with COVID-19 often experience severe cytokine storm syndrome, and previous studies have confirmed that ORF8 contributes to promoting the release of inflammatory cytokines by activating IL-17 pathway (16). Notably, the interleukin IL17RA receptor can contribute to eliminating noxious stimuli to participate in host defense. Still, excessive activation of the IL17RA signaling pathway can promote the expression of inflammatory factors, resulting in the development and progression of inflammatory illnesses (17). SARS-CoV-2 ORF8 can strongly bind to IL17RA, a key receptor in the host cell IL-17 signaling pathway (14). The binding of SARS-CoV-2 ORF8 to IL17RA causes the excessive release of cytokine, leading to the occurrence of the cytokine storm. Additionally, MHC-I enables the host immune system to detect and eliminate foreign antigens, playing an immunomodulatory role during viral infection (18). The SARS-CoV-2 ORF8 has evolved the ability to subvert antigen presentation by downregulating cell surface expression of MHC-I. The structure of SARS-CoV-2 ORF8, containing an Ig-like domain, allows it to bind to MHC-I, potentially mediating immune suppression and evasion activities (16). There are also reports indicating that the virus utilizes binding to ADAM9 receptors, leading to upregulation of ADAM9 expression and affecting the host immune responses during viral infection (19). ADAM9 is a host dependency factor mediating SARS-CoV-2 virus infection and may be an effective molecular target of SARS-CoV-2 ORF8 (15, 20). Despite these above reports, it remains unclear which cytokines or ligands bind to ORF8 to further involve in the host immune regulatory processes. Moreover, it has been shown that ORF8 can modulate innate immune responses by surpassing host interferon-mediated antiviral responses (21). These phenotypic changes by ORF8 may be associated with mutation-induced changes in its structural properties of ORF8 protein (21). Therefore, it is particularly important to further understand the relationship between ORF8’s structure and function. Previously, SARS-CoV-2 ORF8 was obtained by oxidative refolding in Escherichia coli (22). However, it is not clear whether ORF8 expressed from mammalian cells would produce the same structure with the same function.

Here, we determined the crystal structure of SARS-CoV-2 ORF8 expressed from human embryonic kidney cells. The structure of SARS-CoV-2 ORF8 revealed the interesting difference from the structure folded in E. coli. The newly solved structure of SARS-CoV-2 ORF8 provides a foundation for further our understanding its immune-mediated function, including identifying potential binding sites of immunomodulatory molecules, as well as a potential new mechanism for immune evasion.

## RESULTS

### The overall structure of ORF8.

**FIG 1:**
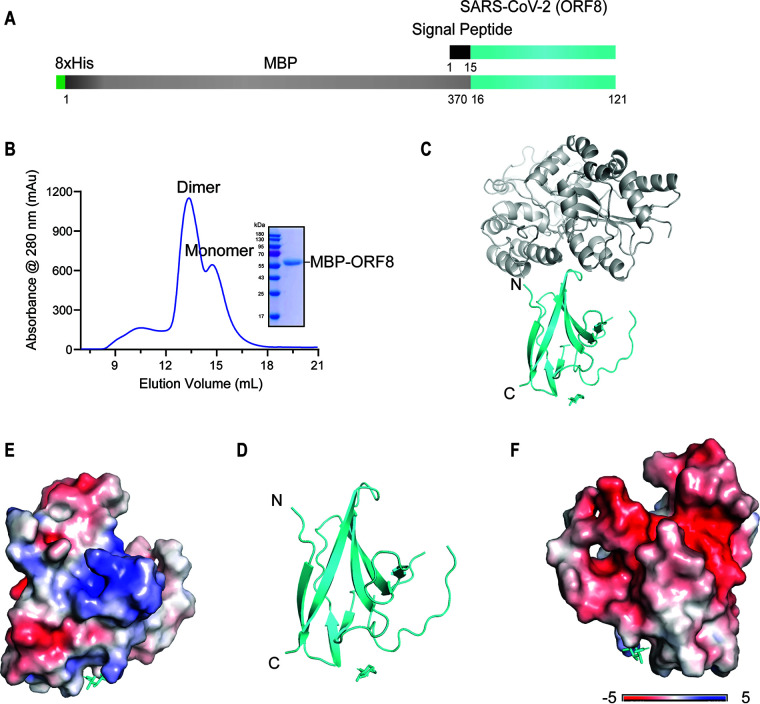
The overall structure of SARS-CoV-2 ORF8. (A) Constructs of SARS-CoV-2 ORF8 used in this study are shown. (B) The purification profile from size exclusion chromatography on a Superdex 200 column and SDS-PAGE results are displayed. The calculated molecular weight of MBP-OFR8 is 53.5 kDa. (C) A cartoon representation of an MBP-ORF8 monomer containing the N-terminal MBP (gray color) and C-terminal ORF8 (cyan color) is shown. (D) A cartoon representation of the detailed view of the ORF8 monomer containing the major six β-strands is displayed. (E and F) The surface electrostatic potential at both front and back sides of each monomer at neutral pH is presented, with positive, negative, and neutral charges shown in blue, red, and white, respectively.

We expressed ORF8 in mammalian cells to obtain ORF8 in its native environment, unlike previous studies which refolded ORF8 in E. coli (22). The nucleotide sequences of SARS-CoV-2 ORF8 were codon-optimized, and an N-terminal 8×His tag was added for purification, and an N-terminal maltose binding protein (MBP) tag was added to facilitate its folding (Fig. 1A). The ORF8 gene was then cloned into a mammalian cell expression vector and transiently expressed in 293F cells. The supernatant was purified with Ni Sepharose excel and size exclusion chromatography (SEC) to high purity. Based on the peak elution volume during the analysis of SEC (Fig. 1B), two peaks represented the dimeric and monomeric forms of ORF8, respectively. After many cycles of crystals screening and optimizations, well-diffracting crystals were obtained, diffracting to a resolution of 2.3 Å with space group C222_1_, and refined to R_work_ and R_free_ at 21.49% and 24.53% respectively (Fig. 1C and Table 1). The ORF8 protein was crystallized in a dimeric form. Each monomer consists of six β-sheets, with two antiparallel β-sheets and one parallel β-sheets. The overall structure exhibits an Ig-like fold (Fig. 1D). The surface of ORF8 is observed to be full of positive charges on one side (Fig. 1E) and negative charges on the other side (Fig. 1F). This indicates that ORF8 adapts different surface electrostatic sides to interact with its binder.

**TABLE 1:** Data collection, structure solution, and refinement statistics

Data set	PDB 7XMN
Data collection	
Space group	*C222_1_*
Unit cell parameters	
*a, b, c* (Å)	140.09, 146.69, 74.72
*a, b, g* (°)	90, 90, 90
Resolution range (Å)	101.31 to 2.30 (2.38 to 2.30)^*a*^
Unique reflections	33261 (3311)
Multiplicity	4.4 (4.5)
Completeness (%)	96.8 (99.2)
*R_merge_* (%)	13.0 (76.2)
*R_pim_* (%)	6.8 (40.2)
*I / s*(*I*)	7.9 (2.3)
CC1/2 of highest resolution shell	0.726
Structure solution and refinement	
Resolution (Å)	60.13 to 2.30 (2.38 to 2.30)
No. of reflections	33249
Completeness (%)	96.11(98.97)
Non-hydrogen atoms	3824
*R_work_ / R_free_* (%)	21.49/24.53 (28.34/29.37)
R.m.s. deviations	
Bond lengths (Å)	0.0025
Bond angles (°)	0.570
*B*-factors (Å^2^)	
Overall	40.33
Protein	40.23
Water	34.51
Other	51.61
Ramachandran plot outliers (%)	0.00
Molprobity score	1.05
Molprobity clashscore	1.90

a Values in parentheses are for the highest resolution shell.

### Four pairs of disulfide bonds stabilize the ORF8 fold.

**FIG 2:**
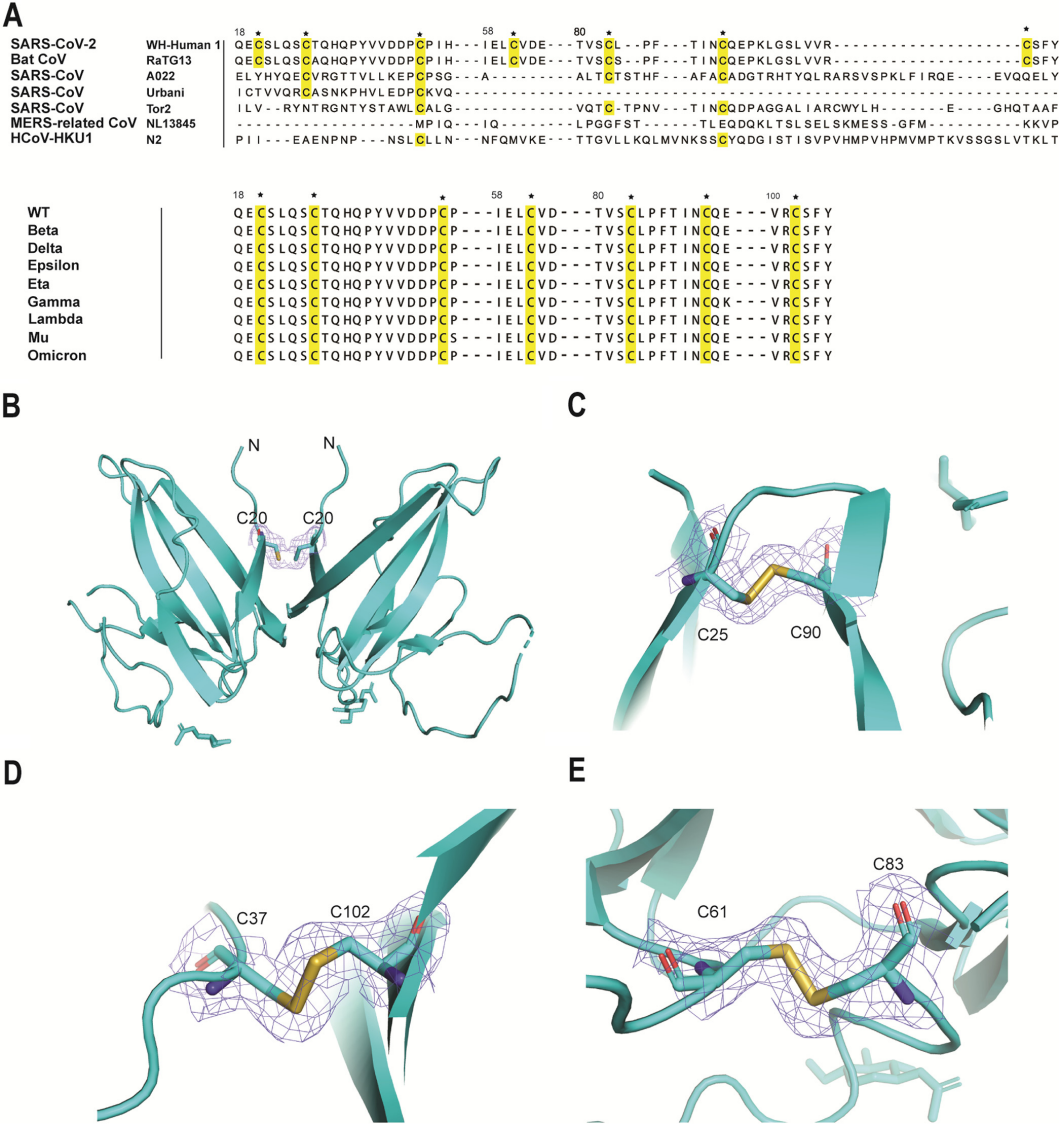
Four pairs of disulfide bonds stabilize the Ig-like fold of SARS-CoV-2 ORF8, forming a stable dimeric structure. (A) Alignment of SARS-CoV-2 OFR8 with related coronavirus strains (top), and alignment of SARS-CoV-2 ORF8 with another variant (bottom). The conservation analysis of primary sequences involved in the formation of pairs disulfide bonds in ORF8 is shown. Pentagrams indicate residues that form disulfide bonds, and their conservation is highlighted in yellow. The homologs involved in the alignment include bat-type coronavirus strains related to SARS-CoV-2 ORF8 and human-infecting related coronavirus strains. The UniProt or GISAID identification numbers for the sequences used in this section are as follows. SARS-CoV-2 (WT): P0DTC8; Bat CoV RaTG13: A0A6B9WE90; SARS-CoV A022: Q4JDR1; SARS-CoV Urbani: Q7TFA0; SARS-CoV Tor2: Q80H93; MERS-related CoV: A0A2R4KP81; HCoV-HKU1: P0C5B5. Beta: EPI_ISL_5416540; Delta: EPI_ISL_2001211; Epsilon: EPI_ISL_1020315; Eta: EPI_ISL_1093470; Gamma: EPI_ISL_6228367; Lambda: EPI_ISL_11298199; Mu: EPI_ISL_6526278; Omicron: EPI_ISL_6699751. (B) to (E) The cartoon model sequentially shows a pair of intermolecular and 3 intramolecular disulfide bonds, and the residues involved in the formation of disulfide bonds are shown separately. The 2Fc-Fo electronic density map with 2 in the disulfide bond region is shown in blue color.

To compare the variation during the rapid evolution of ORF8, six coronaviruses with various host closely related to SARS-CoV-2 were selected for alignment. Among the several coronaviruses that are pathogenic to humans: Bat CoV RaTG13 is considered to have closely related ORF8 homologs with 92.86% of nucleotide similarity to SARS-CoV-2 (23), SARS-CoV and MERS-CoV are associated with severe acute respiratory syndrome in humans, like SARS-CoV-2, showing 79% and 50% nucleotide similarity to SARS-CoV-2, respectively, HCoV-HUK1 is one of four β-CoV that normally cause cold symptoms in humans (24). It should be noted that the intact ORF8 in early human isolates encoded 122 amino acids, which is SARS-CoV A022. But in late strains, the 29-nucleotide deletion resulted in ORF8 being split into ORF8a and ORF8b, which are SARS-CoV Urbani and SARS-CoV Tor2, respectively (25). The diversity of the ORF8 proteins may be attributed to its stability. Using a mammalian cell expression, the ORF8 protein was expressed in soluble, and we observed four pairs of disulfide bonds (3 intramolecular and 1 intermolecular disulfide bonds) from the crystal structure, predicting that SARS-CoV-2 ORF8 forms a stable dimeric structure. Multiple sequence alignment of ORF8 proteins in Bat CoV RaTG13, SARS-CoV, MERS-CoV and HCoV-HUK1 shows the conservation of all 7 cysteine residues between SARS-CoV-2 ORF8 and Bat CoV RaTG13 ORF8 (Fig. 2A). All 7 cysteine residues were also conserved across different strains of SARS-CoV-2 ORF8. The dimeric ORF8 is linked by an intermolecular disulfide bridge formed between each monomer of C20 (Fig. 2B). However, C20, responsible for the intermolecular disulfide bond, is not found in other human-infecting coronavirus strains, indicating that if the functional form is dimer for all strains, Bat CoV RaTG13 and SARS-CoV 2 would have the most stable dimeric ORF8, as supported by *in-silico* mutagenesis computation (Table S2-S9). Furthermore, SARS-CoV-2 ORF8, like CoV RaTG13 ORF8, has 3 intramolecular disulfide bonds, including C25 and C90, C37 and C102, C61 and C83, respectively (Fig. 2C-E). Not all these intramolecular disulfide bonds were found in SARS-CoV, MERS-CoV or HCoV-HKU1. Thus, SARS-CoV-2 and Bat CoV RaTG13 ORF8 would have the most stable monomeric structure as well.

### The ORF8 protein is a glycosylated protein.

Glycosylation modification is reported to impact the viral cycle in many stages (26). Viruses utilize host cell machinery to glycosylate their proteins during replication, thereby masking potentially immunogenic epitopes. Furthermore, pathogens can exert immune evasion and enhance immune cell infection through glycan shielding (27). In addition to O-glycosylation, N-glycosylation is one of the most common post-translational modifications for proteins. The modification of proteins by N-glycosylation plays an essential role in many physiological and pathological processes, such as protein folding, transport, signal transduction (28), and cooperatively control of T cell function (29). Based on this, we focused on analyzing the glycosylation characteristics of ORF8.

**FIG 3:**
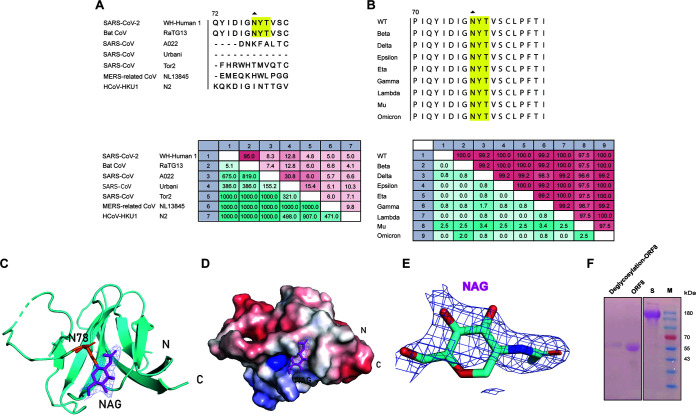
Glycosylation modification at position N78 of SARS-CoV-2 ORF8. (A and B) The conservation analysis of glycosylation motif at positions 78 to 80 of several CoVs is highlighted with triangles and yellow. (A and B) Upper panels show the alignment among related coronavirus (left) and different SARS-CoV-2 variants (right). (A and B) Lower panels show the pairwise comparison matrix in related coronavirus (left) and different SARS-CoV-2 variants (right). The numbers above the diagonal line in the matrix are the percent identity between the 2 viruses, and the below are the divergence in residues composition between the 2 viruses. The shade of color indicates the degree of difference. (C) A detailed view of the glycosylation at position N78 of ORF8 colored in red is shown. The establishment of a cartoon model of its glycosylation is resolved by fitting the NAG oligosaccharide ring into the 2F0-Fc map with 2 colors in blue. (D) The surface electrostatic potential of the region centered on glycosylation at position N78 at neutral pH is shown. The NAG glycosylated ring, N-terminus, and C-terminus are shown separately. (E) The electron density omits map of NAG. (F) Glycan staining identification plots for deglycosylated and glycosylated modified ORF8 are shown (from left to right: Deglycosylated ORF8, ORF8, S protein, and Marker).

Given the importance of glycosylation, the glycosylated residue motif of ORF8 from different betacoronaviruses has been predicted, possible glycosylated residues are N78, Y79, and T80, which are conserved between SARS-CoV-2 and bat RaTG13 ORF8, as well as in different SARS-CoV-2 variants, but not in the residues for other coronaviruses (Fig. 3A and 3B). As expected, the structure of SARS-CoV-2 ORF8 we determined contains an N-glycosylation modification linked to residue N78 with clear N-acetyl-beta-d-glucosamine (NAG) electronic density (Fig. 3C). The NAG glycosylation covers a neutral surface but is close to a positively charged pocket (Fig. 3D), and the NAG's electron density omit map shows a good fitting (Fig. 3E). The presence of ORF8 glycan modification was also identified by Glycogen Periodic acid-Schiff Staining Kit, and ORF8 treated with deglycosylation enzyme PNGase F could not be stained (Fig. 3F). The differences in glycosylation modification of ORF8 for various CoVs indicates a possible effect on the impact of the viral antigenicity or pathogenicity (30).

### The ORF8 protein is a lipid-binding protein.

**FIG 4:**
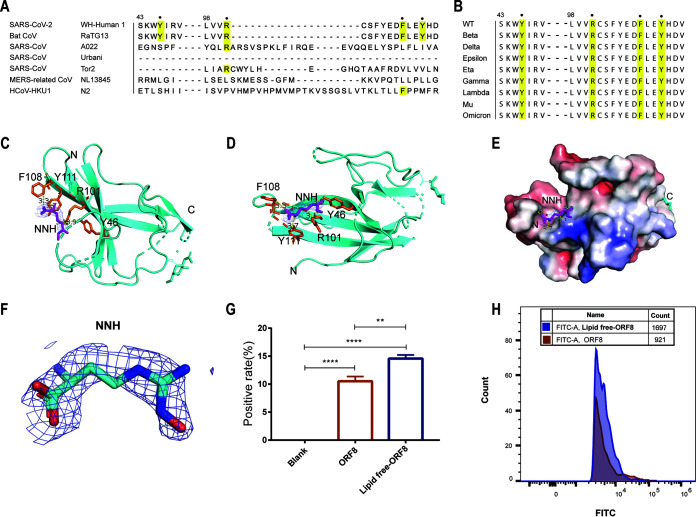
SARS-CoV-2 ORF8 is a lipid-binding protein. (A and B) The primary sequence alignment of key residues involved in lipid-binding pocket formation in related coronaviruses (A) and different variant strains (B) is highlighted with the solid dots and yellow. (C) The lipid-binding pocket is mainly composed of 4 residues Y46, R101, F108, and Y111, and is filled with lipids which are fitting with NNH in the 2Fo-Fc map with 2. Key residues involved in the lipid-binding pocket are shown in orange, and NNH as the inserted lipid is shown in purple. The distance between the lipid and the pocket is shown to be 3.3, 3.7, 3.1 and 3.9 Å to F108, Y111, R101, and Y46, respectively. (D) The model is shown flipped so that (C) is visible. (E) The surface electrostatic potential of the region centered on the lipid-binding pocket is shown at neutral pH, and the distance between the inserted lipid and the pocket is shown. (F) The electron density omit map of NNH is displayed. (G) Bar graphs of ORF8 and lipid-free ORF8 binding to immune monocytes are presented. (H) The superimposed contrast d histogram of ORF8 and lipid-free ORF8 binding to immune monocytes. The ** symbol indicates *P* < 0.01, while the*** symbol indicates *P* < 0.001.

Lipids often interact with proteins to regulate protein localization and activity. Viral proteins utilize fatty acids from the host cell for lipidation, thereby stabilizing their protein structures and directly affecting their spatial distribution and dynamics. Differences in residues composition may give rise to various lipid selectivity in proteins (31). The greater the diversity of residues, the more noticeable the changes in lipid-binding sites. Therefore, we performed an alignment of SARS-CoV-2 ORF8 with several closely related coronaviruses. From the alignment results of key residues formed by the lipid-binding pockets, except that Bat CoV RaTG13, which has a highly consistent residues with SARS-CoV-2, other coronaviruses do not have the conservation of lipid-binding regions (Fig. 4A). SARS-CoV urbani and SARS-CoV Tor2 also showed significant diversity with the key residues of SARS-CoV-2 ORF8. Meanwhile, we also aligned the key residues in the lipid-binding region of nine variants in the SARS-CoV-2 family. Interestingly, the residues composing the lipid-binding pocket are largely conserved and all located in the encirclement of Y46, R101, F108, and Y111 residues (Fig. 4B). In contrast to the previously reported SARS-CoV-2 ORF8 structure, we observed that the ORF8 expressed in 293F cells was a lipid-binding protein with a lipid-binding pocket. The lipid-binding pocket is a hydrophobic region surrounded by four residues Y46, R101, F108, and Y111. The distances from the lipid to four residues R101, F108, Y111, and Y46 are 3.1 Å, 3.3 Å, 3.7 Å, and 3.9 Å, respectively (Fig. 4C and D). Observing the structural details of SARS-CoV-2 ORF8, we found that there was electronic density occupied by a curved pocket in the hydrophobic region. The bottom view of the structure showed that the lipid occupies the cavity consisting of four residues, forming a stable hydrophobic core region (Fig. 4D). Similarly, the distance between the inserted lipid and the pocket is shown on the surface with electrostatic potential, exhibiting the relative positions of the fatty acids (Fig. 4E). This electronic density region is highly consistent with nor-n-omega-hydroxy-l-arginine (NNH) fatty acids, which can almost fit into the pocket entirely (Fig. 4F). The above observations are also consistent with the results of alignment and the divergence in amino acid composition.

Covalent lipid modification of proteins can alter the physical and chemical properties and thus affect their physiological functions. Protein lipidation modifications may induce structural changes by interacting with cell membranes and participating in the signal transduction of host cells (32). The presence or absence of lipid sites can help us better understand the role of structural changes in ORF8 in viral evolution. To further examine the effect of the presence or absence of lipids on the involvement of ORF8 protein in human immune regulation, we separately co-incubated the delipidated ORF8 and ORF8 protein in the presence of lipid modifications with human PBMC cells. We found that the delipidated ORF8 protein had a higher percentage of binding to human immune cells (Fig. 4G and H), suggesting that the lipid modification of ORF8 inhibits its binding to human immune cells and thus promotes the immune escape effect of the virus in humans.

### Three major loops contribute to immune regulation.

**FIG 5:**
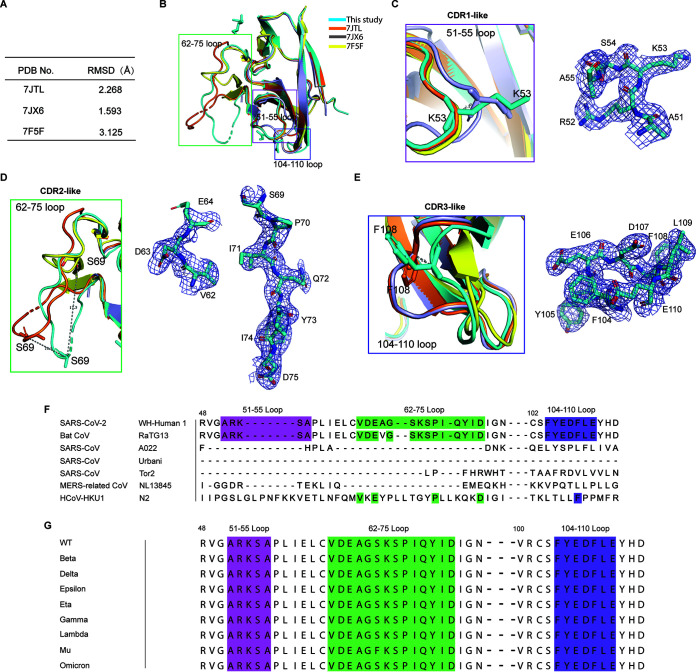
SARS-CoV-2 ORF8 contains three functional loops. (A) The RSMD values were tallied after comparing the previously obtained structures (7JTX, 7JX6, and 7F5F) with the present study. (B) The ORF8 structure was compared between our ORF8 structure and structures from PDB ID: 7JTL, 7JX6, and 7F5F. (C to E) A detailed view of the three functional loops with their closely related structural alignments and electron density omit map is presented. The distance between S69 in the 62 to 75 loop and S69 in 7JTL and 7F5F is 10.3 Å and 17.3 Å, respectively. The distance between K53 in 51 to 55 loop and K53 in 7JX6 is 2.3 Å. The distance between F108 in the 104 to 110 loop and F108 in 7JTL is 2.9 Å. (F and G) The alignment of the primary sequences of the three loops in related coronaviruses and different variant strains are highlighted in fuchsia, green, and purple, respectively.

We aligned the structure determined in this study with previous obtained structures (7JTX, 7JX6, and 7F5F) and found that the overall RMSD at 2.268 Å, 1.593 Å, and 3.125 Å respectively (Fig. 5A). Large diversity is visible, mainly located in the regions of residues 51–55, 62-75, and 104–110, named as CDR1-like, CDR2-like, and CDR3-like domains, respectively (Fig. 5B-E). For example, the distance between S69 in the 62–75 loop in the structure of this study and S69 in 7JTL and 7F5F is 10.3 Å and 17.3 Å, respectively. The distance between K53 in the 51–55 loop and K53 in 7JX6 is 2.3 Å. Additionally, the distance between F108 in the 104–110 loop and F108 in 7JTL is 2.9 Å. The three loops in the tertiary structure of soluble ORF8 expressed by the eukaryotic system reflect a large difference from the prokaryotic system, suggesting that the ORF8 protein expressed by the prokaryotic system or obtained by renaturation affects its three-dimensional structure to some extent.

The three loops mentioned above are highly conserved between SARS-CoV-2 and bat RaTG13 ORF8 and among different variants of SARS-CoV-2 (Fig. 5F and G). However, none of the other five related coronaviruses’ ORF8 have the same residues in the three loops as SARS-CoV-2 (Fig. 5F). Remarkably, all nine SARS-CoV-2 variants retained the three loops in ORF8 completely (Fig. 5G). The highly conserved nature of these three loops in ORF8 suggests that they play an critical role in maintaining the stability and the function of ORF8.

Based on the reported potential immunomolecular targets of SARS-CoV-2 ORF8 (13, 14, 18), we examined the possible interactions of SARS-CoV-2 ORF8 with several proteins implicated in immune responses by predicting possible complex structures using AlphaFold2. AlphaFold2 predicts the interaction between SARS-CoV-2 ORF8 and IL17RA (Fig. S1A) through the CDR1-like and CDR2-like domains of SARS-CoV-2 ORF8, as shown in Fig. S1B. Similarly, SARS-CoV-2 ORF8 was found to form a complex structure with MHC-I (Fig. S1C and D) (33, 34), also through the CDR1-like and CDR2-like domains of SARS-CoV-2 ORF8. ORF8 was also found to be complexed with ADAM9 through the CDR1-like and CDR3-like domains (Fig. S1E and F).

### SARS-CoV-2 ORF8 manifests its role in human immunity by interacting with human monocytes.

**FIG 6:**
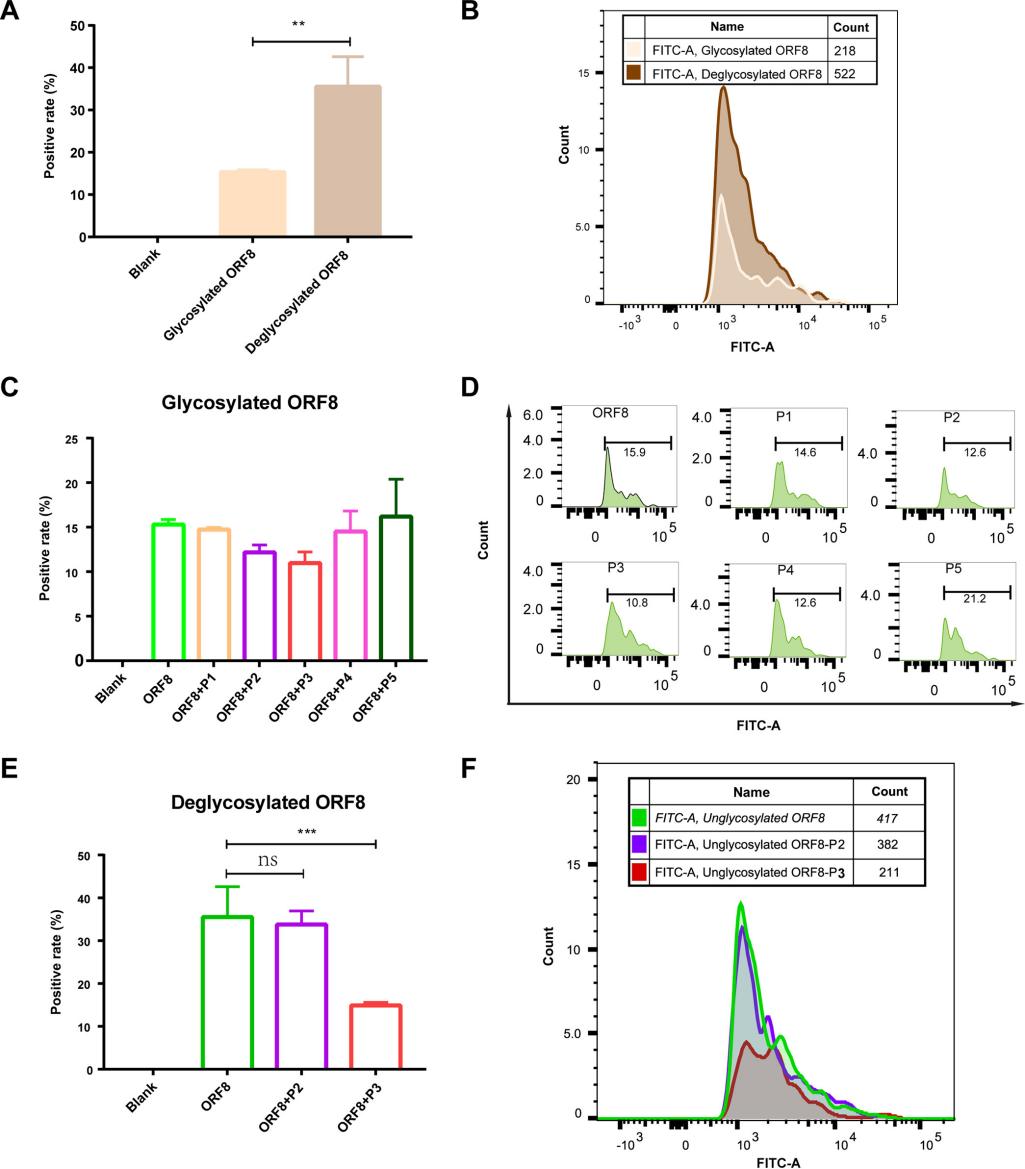
SARS-CoV-2 ORF8 manifests its role in human immunity by interacting with human monocytes. (A) Bar graphs of glycosylated ORF8 and deglycosylated ORF8 binding to immune monocytes are shown. (B) The superimposed contrast histogram of glycosylated ORF8 and deglycosylated ORF8 binding to immune monocytes is presented. (C) A bar chart of the inhibitory effect of 5 peptides on the binding of glycosylated ORF8 to immune monocytes is shown. (D) The histogram of the inhibitory effect of the corresponding 5 peptides on the binding of glycosylated ORF8 to immune monocytes is presented. (E) A bar graph of the inhibitory effect of peptide 1 and peptide 2 on the binding of the deglycosylated ORF8 to immune monocytes is shown. (F) The histogram of the inhibitory effect of the corresponding P1 and P2 on the binding of deglycosylated ORF8 to immune monocytes is presented. ns indicates no statistical difference; ** indicates *P* < 0.01, and *** indicates *P* < 0.001.

To validate the interaction interface of IL17RA, MHC-I, and ADAM9 with ORF8 based on the prediction results of AlphaFold2, we designed five peptides against ORF8. Information about these peptides is listed in Table S1. We performed SPR affinity assays of these five peptides with ORF8, and the results indicated that all five peptides can bind to ORF8 at the micromolar level of binding affinity (Fig. S2). Previous studies have shown that ORF8 is identified as a new immune target of the immune response that involves host immune regulation in SARS-CoV-2 (35). ORF8 antibody has been used as accurate serological marker for early and late SARS-CoV-2 infection, and the tropism of B cells to ORF8 is significantly increased in patients with COVID-19 (7, 35). To examine whether these peptides can inhibit the binding of coronavirus ORF8 to immune cells, we performed immune cell binding assays. Using peripheral blood mononuclear cell samples, we verified the ability of five peptides to interact with two states of SARS-CoV-2 ORF8 (glycosylation and deglycosylation) with human mononuclear cells. We found that glycosylated ORF8 has a weak binding to immune cells, but deglycosylated ORF8 has a 2-fold stronger binding (Fig. 6A and B). The five peptides did not show significant inhibition of glycosylated ORF8 binding to immune cells (Fig. 6C and D). Interesting, we found that peptide P3, derived from IL17RA, significantly inhibited the binding of deglycosylated ORF8 to immune monocytes (Fig. 6E and F). This further suggests that the glycosylation modification of ORF8 is important for its involvement in human immune regulation. The deglycosylation ORF8 protein can bind to more human immune cells and mobilize the host immune response. The inhibitory effect of peptide P3 on the binding of deglycosylated ORF8 to immune cells suggests that ORF8 likely plays a critical role in human immune regulation through binding to IL17RA.

## DISCUSSION

The COVID-19 pandemic caused by SARS-CoV-2 has created a significant health crisis and posed a huge threat to the global economy. The mutations of SARS-CoV-2 have led to the rapid spread of the virus, which has increased the socioeconomic cost of epidemic prevention and control. This situation has pushes us to seek more strategies for developing treatments. Since SARS-CoV-2 shares phylogenetic traits with related betacoronaviruses, including multiple variants of SARS-CoV-2, it may provide insights into discoverying new antiviral drug targets. Meanwhile, the cytokine storm triggered by viral infection may be related to immune escape mediated by viral involvement. This inspired us to grasp the molecular details of viral involvement in immune regulation from a structural perspective. ORF8 is a rapidly evolving accessory protein with the least similarity to other betacoronaviruses. In this study, we expressed and purified the SARS-CoV-2 ORF8 protein in a mammalian cell and determined its crystal structure by X-ray crystallography. Many new features were identified in this structure, and further validation was done in immune cells, which provides more evidence to explore its function in immune regulation.

One of the key findings from the SARS-CoV-2 ORF8 structure is the observation of four pairs of disulfide bonds. An intermolecular disulfide bridge formed by each monomer of C20 residues can be observed in the SARS-CoV-2 ORF8 structure. The intramolecular disulfide bonds are conserved among SARS-CoV-2 variant, but there are certain differences among other betacoronaviruses, especially several other human-infecting coronavirus strains. Intermolecular and intramolecular disulfide bonds play an essential role in maintaining a stable dimer structure of SARS-CoV-2 ORF8.

We also found the N78 residue of ORF8 protein were glycosylated. Glycosylation modification in viral protein has a wide range of functions, including protein stability and immune evasion. This was confirmed during the experiments where the solubility of ORF8 deteriorated after deglycosylated, further confirming the importance of glycosylation modification for maintaining ORF8 stability. Moreover, the presence or absence of glycosylation modification at position N78 is directly related to the residue divergence of different coronavirus or SARS-CoV-2 variants. The new finding of glycosylation at N78 residue may contribute to explaining the highly pathogenicity for SARS-CoV-2. In immune cell experiments, we also found that the binding ability of glycosylated ORF8 to immune cells was weakened, providing an explanation for the contribution of glycosylation-modified ORF8 in viral immune escape, which is similar to previously reported obsevations (11).

From the SARS-CoV-2 ORF8 structure, we found that ORF8 is a lipid-binding protein with a hydrophobic region surrounded by four residues: Y46, R101, F108, and Y111. The lipid-binding pockets were also formed by the residues of SARS-CoV-2 variants but not in other betacoronaviruses. The ORF8 protein may be lipidated by the host cell, thereby stabilizing its protein structure and directly affecting its spatial distribution and dynamics. At the same time, this lipid hydrophobic cavity also provides potential regulation for immune evasion.

Notably, SARS-CoV-2 ORF8 contains three functional loops, CDR1-like, CDR2-like, and CDR3-like domains, that form Ig-like folded domains. These domains may evade host immune system monitoring by interacting with immunomodulatory receptors IL17RA, MHC-1, and ADAM9 (36). Similar to this study, recent investigation found that ORF8 can disrupt host epigenetic regulation via its CDR1-like domain. From the perspective of immune regulation, identifying host-dependent factors that mediate viral infection by SARS-CoV-2 ORF8 may provide key insights to identify the targets of the immune response during SARS-CoV-2 infection.

In the immune cell assay, the reason for the weaker binding ability of glycosylated ORF8 to immune cells than non-glycosylated has not been clearly reported yet. We speculate that the reason may be related to the modification of ORF8 glycosylation, which would directly or indirectly spatially impede the binding surface of ORF8 to immune cytokines. It is worth mentioning that we found that only in the deglycosylation ORF8 binding assay with immune cells could present a significant inhibitory effect of peptide P3, while in the glycosylated ORF8, there was no significant inhibitory effect of peptide P3, but there was a tendency to inhibit the immune cell. This may be related to the low percentage of ORF8 binding to immune cells after glycosylation.

This study has several limitations that could be addressed in future research. Firstly, while we observed glycosylation in the crystal structure of ORF8 and validated its involvement in immune cell regulation, we lack clinical evidence demonstrating how this glycosylation regulates immune function. Further studies could investigate this aspect to provide a more comprehensive understanding of ORF8's role in immune regulation. Secondly, our identification of a lipid-binding pocket in ORF8 requires further confirmation through additional experiments. Specifically, it would be valuable to determine which types of lipids bind to ORF8 and how this interaction mediates immune cell function. Lastly, although we identified the CDR-like domains as potential mediators of ORF8-binder interactions, the structure of the ORF8/binder complex remains unknown. Future research could focus on solving the structure of this complex to provide further insights into ORF8's interactions with its binding partners.

In a word, this study reveals several new features of ORF8, like glycosylation modification, lipid-binding and three CDR-like domains, which may contribute to a deeper understanding of the mechanism of immune evasion for SARS-CoV-2 infection.

## MATERIALS AND METHODS

### Constructs.

The sequence encoding residues 16–121 of the SARS-CoV-2 ORF8 (GenBank ID: 43740577) was optimized for codon usage and subsequently subcloned into the pHLmBMP-3 vector (37). The resulting recombinant plasmids were sequenced and verified prior to use.

### Protein expression and purification.

The protein was transiently expressed in high-density human embryonic kidney 293F cells at 37°C and 5% CO_2_ to ensure proper folding and post-translational modification. Transient transfection was carried out at a density of approximately 4–8 × 10^6^ cells/mL using a 1:3 mass ratio of expression plasmid to transfection reagent polyethyleneimine (PEI). After transfection, the cells were cultured in an orbital shaking incubator at a speed of 130 rpm for 5–6 days. The target protein was purified from cell expression supernatant using Ni Sepharose excel. Gradient washing with a pH 7.5 buffer containing 20 mM Tris-HCl and 250 mM NaCl with increasing concentrations of imidazole, was followed followed by elution of the target protein with 250 mM imidazole. The eluted solution containing the target protein was loaded onto a size exclusion column (Superdex 200, Cytiva) for further purification. The Superdex 200 column was eluted with the same pH 7.5 buffer containing 20 mM Tris-HCl and 150 mM NaCl for 1.2 column volumes. Finally, the protein purity was determined by SDS-PAGE, and the protein was concentrated to 36 mg/mL and stored at −80°C. To produce deglycosylated ORF8 protein, the protein was treated with PNGase F enzyme overnight before loading onto Superdex 200 column. To produce liplid-free ORF8 protein, the protein was treated with 0.5% Trition X-100 overnight before loading onto Superdex 200 column.

### Crystallization.

The initial crystallization conditions for the protein crystals were obtained using Hampton Research Kits and a protein concentration of 15 mg/mL in 72-well by microbatch plates. The initial hit conditions were then optimized using homemade buffers. The optimized solution (1 μL) was then mixed with 1 μL of the protein and 0.2 μL of seeds. Crystals appeared after approximately 3 months of growth at 18°C, followed by continued growth in 24-well hanging drop plates for another 3 months. The protein concentration was then increased to 36 mg/mL to accelerate the crystal growth. The final crystal growth conditions were 0.1 M MES pH 6.1, 2% vol/vol Polyethylene glycol 400, and 2.1 M Ammonium sulfate. The final crystals were vitrified in a crystallization solution containing 25% glycerol and flash-frozen in liquid nitrogen.

### Structure determination and analysis.

X-ray diffraction data were collected at beamline stations BL18U1, BL02U1, and BL19U1 of Shanghai Synchrotron Radiation Facility (SSRF), China. The distance from the center of the crystal to the detector was 400 mm, and the exposure time was 0.2 s with no attenuation value set. One image per degree (360 images) was collected. The data were processed using XDS (38), followed by molecular replacement using PDB 3SET as the searching model with Phaser (39). Model rebuilding was carried out using buccaneer, and refinement and manual model modification were performed using Refmac and Phenix (40) in Coot (41). The placement of water molecules was performed after the refinement of atomic positions and B-factors. The figures were produced using PyMOL. Table 1 shows the statistics of data collection and refinement. The electron density omit maps were generated using Phenix and produced using PyMOL.

### Glycan staining.

To analyze glycosylation modifications, ORF8 protein was first deglycosylated using PNGase F at a molar ratio of 1:100. Subsequently, ORF8, deglycosylated ORF8, the S protein of SARS-CoV-2 (Glycosylated protein), and an unrelated protein (not shown) were loaded onto SDS-PAGE at a loading mass of 10 μg and transferred to PVDF membrane. Finally, glycosylation modifications were determined using the Glycogen Periodic acid-Schiff Staining (PAS) Kit (Thermo Scientific).

### Bioinformatics and design of peptides.

*In-silico* mutagenesis were predicted using CUPSAT server (http://cupsat.tu-bs.de/index.jsp). The complex structures of ORF8/IL17RA, ORF8/MHC-I, and ORF8/ADAM9 were predicted using the standalone AlphaFold 2.2 soft package with its multiple functions. The models were analyzed on PISA server (https://www.ebi.ac.uk/msd-srv/prot_int/cgi-bin/piserver) and manually checked in PyMOL. Based on the predicted results, we designed five peptides for subsequent experiments by analyzing the interaction interfaces of ORF8/IL17RA, ORF8/MHC-I, and ORF8/ADAM9.

### Surface plasmon resonance (SPR).

Five peptides (P1-P5) were synthesized by GenScript (Nanjing, Jiangsu, China). The purified ORF8 protein (>95% purity) was immobilized on a 3D Dextran chip. All five peptides were solubilized in PBS buffer at concentrations of 10 μM, 5 μM, 2.5 μM and 1.25 μM, respectively. The chips were washed with PBS and regeneration buffer (10 mM Glycin-HCl pH 2.0) before running the setup program on a PlexArray HT high-throughput intermolecular interaction screening instrument (Plexera, United States). The binding and dissociation times were both set to 300 s. The raw data were processed and calculated using BIAevaluation software and plotted using origin 8.0.

### Immune cell binding assay involving peptides.

The purified recombinant SARS-CoV-2 ORF8 protein was completely deglycosylated using PNGase F enzyme. Both the glycosylated ORF8 and the deglycosylated ORF8 protein were subsequently labeled with FITC. Human peripheral blood mononuclear cells (PBMCs) were isolated from the peripheral blood of healthy donors using the human peripheral blood lymphocyte separation solution-FICOLL (Beyotime, China). The extracted PBMC cells were incubated for 2 h, and then the suspended lymphocytes were removed. Next, 50 μM peptide with 5 μM labeled ORF8 protein was added to monocytes in each well after incubation on ice for 2 h. Cells were collected after 4 h of incubation in a 37°C incubator and incubated with human FcRs blocker (BioLegend, United States) on ice for 20 min. Subsequently, the cells were terminated with PBS and washed twice. The binding efficiency was then determined by flow cytometry (Beckman, United States) and the data were processed using Flowjo_8.1 software.

### Data availability.

The structure obtained in this study has been deposited in the Protein Data Bank with the ID: 7XMN.
