# Supplementary material for: Glycosylated, Lipid-Binding, CDR-Like Domains of SARS-CoV-2 ORF8 Indicate Unique Sites of Immune Regulation
Source: Microbiol Spectr. 2023 Jun 15;11(4):e01234-23. doi: 10.1128/spectrum.01234-23 (PMC10434001; doi:10.1128/spectrum.01234-23)
Supplement: Supplemental file 1 — Supplemental material. Download spectrum.01234-23-s0001.pdf, PDF file, 1.1 MB [file spectrum.01234-23-s0001.pdf]

Glycosylated, lipid-binding, CDR-like domains of SARS-CoV-2 ORF8 indicate unique sites of immune regulation

Fang Wu[a,†], Xin Chen[a,†], Yanhong Ma[a,†], Yuzhe Wu[a], Rui Li[a], Yuanwei Huang[a], Rong Zhang[c], Yaoqi Zhou[b,*], Jian Zhan[b,*], Shuwen Liu[a,*], Wei Xu[a,*]

[a] Guangdong Provincial Key Laboratory of New Drug Screening, School of Pharmaceutical Sciences, Southern Medical University, Guangzhou, 510515, China.

[b] Institute for Systems and Physical Biology, Shenzhen Bay Laboratory, Shenzhen, Guangdong, 518038, China.

[c] Key Laboratory of Medical Molecular Virology (MOE/NHC/CAMS), School of Basic Medical Sciences, Shanghai Medical College, Biosafety Level 3 Laboratory, Fudan University, Shanghai, 200433, China.

*Corresponding author

E-mail addresses: xuwei3322@smu.edu.cn (W.X), liusw@smu.edu.cn (S.L), zhanjian@szbl.ac.cn (J.Z), zhouyq@szbl.ac.cn (Y.Z)

†These authors contributed equally.

Figure S1

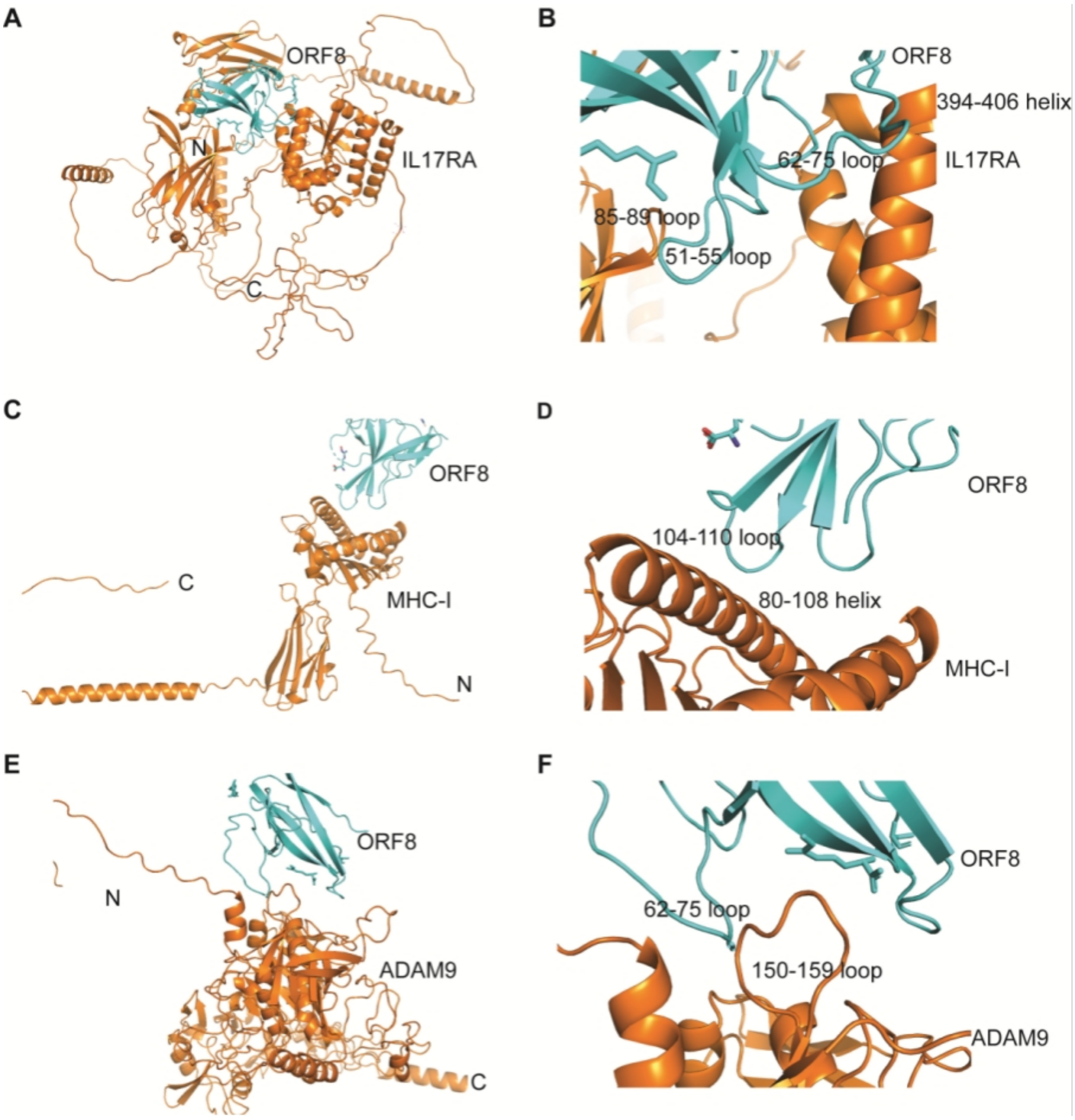

Figure S1 AlphaFold2 predicts the interaction between SARS-CoV-2 ORF8 and IL17RA, MHC-I, or ADAM9. (**A**) AlphaFold2 predicts the interaction between ORF8 and IL-17RA. IL 17RA is shown in orange, and ORF8 is shown in cyan. (**B**) A detailed view of IL17RA and ORF8 interaction interface. The 51-55 loop and 62-75 loop of ORF8 are labeled to interact with IL17RA. (**C**) AlphaFold2 predicts the interaction between ORF8 and MHC-I. MHC-I is shown in orange, and ORF8 is shown in cyan. (**D**) A detailed view of MHC-I and ORF8 interaction interface is presented, and the 62-75 loop and 104-110 loop of ORF8 are labeled to

interact with MHC-I. (**E**) AlphaFold2 predicts the interaction between ORF8 and ADAM9. ADAM9 is shown in orange, and ORF8 is shown in cyan. (**F**) A Detailed view of ADAM9 and ORF8 interaction interface is presented, and the 51-55 loop and 62-75 loop of ORF8 are labeled to interact with ADAM9.

Figure S2

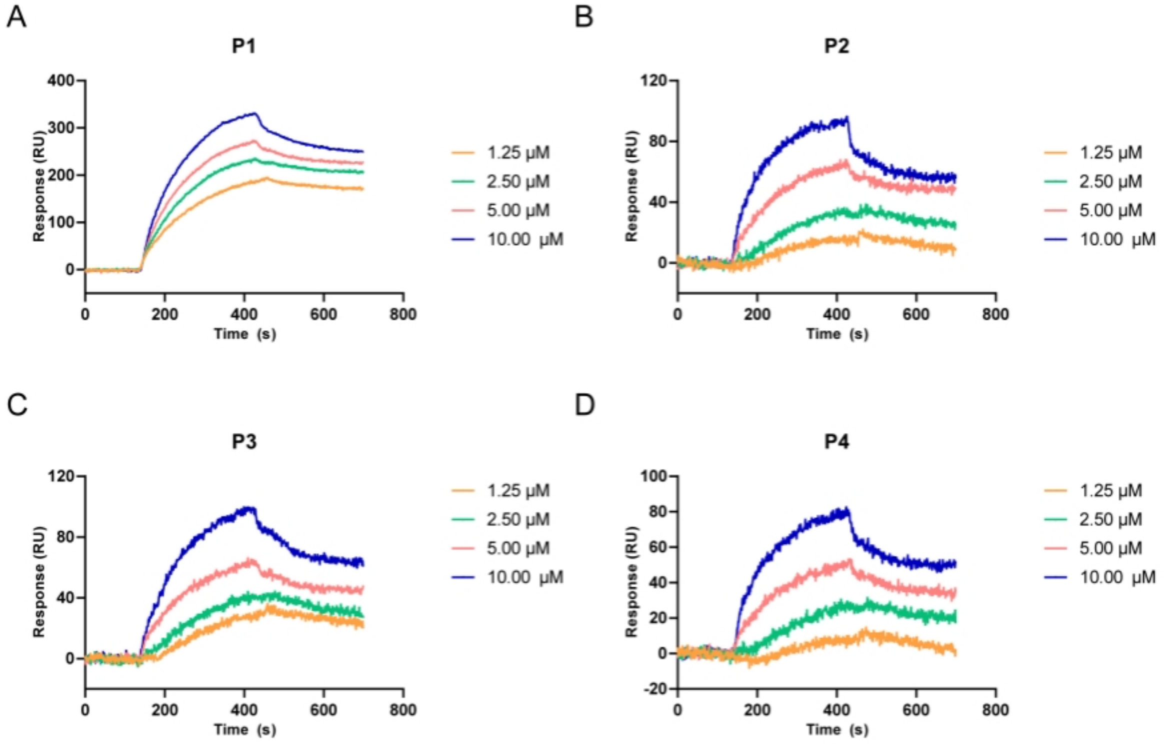

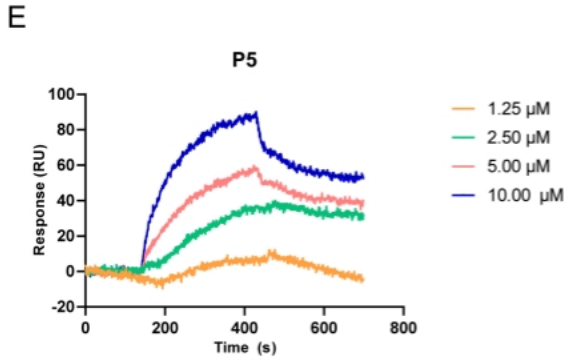

F

Name	K_D
P1	1.79e-7
P2	1.25e-6
P3	8.89e-7
P4	1.51e-6
P5	1.99e-6

Figure S2 The affinity of five peptides (P1-P5) to ORF8 was determined using the SPR method. (**A-E**) The kinetic profiles of five peptides (P1-P5) with ORF8 affinity are shown. (**F**) The statistical results of K_D of 5 peptides (P1-P5) with ORF8 are presented.

Figure S3

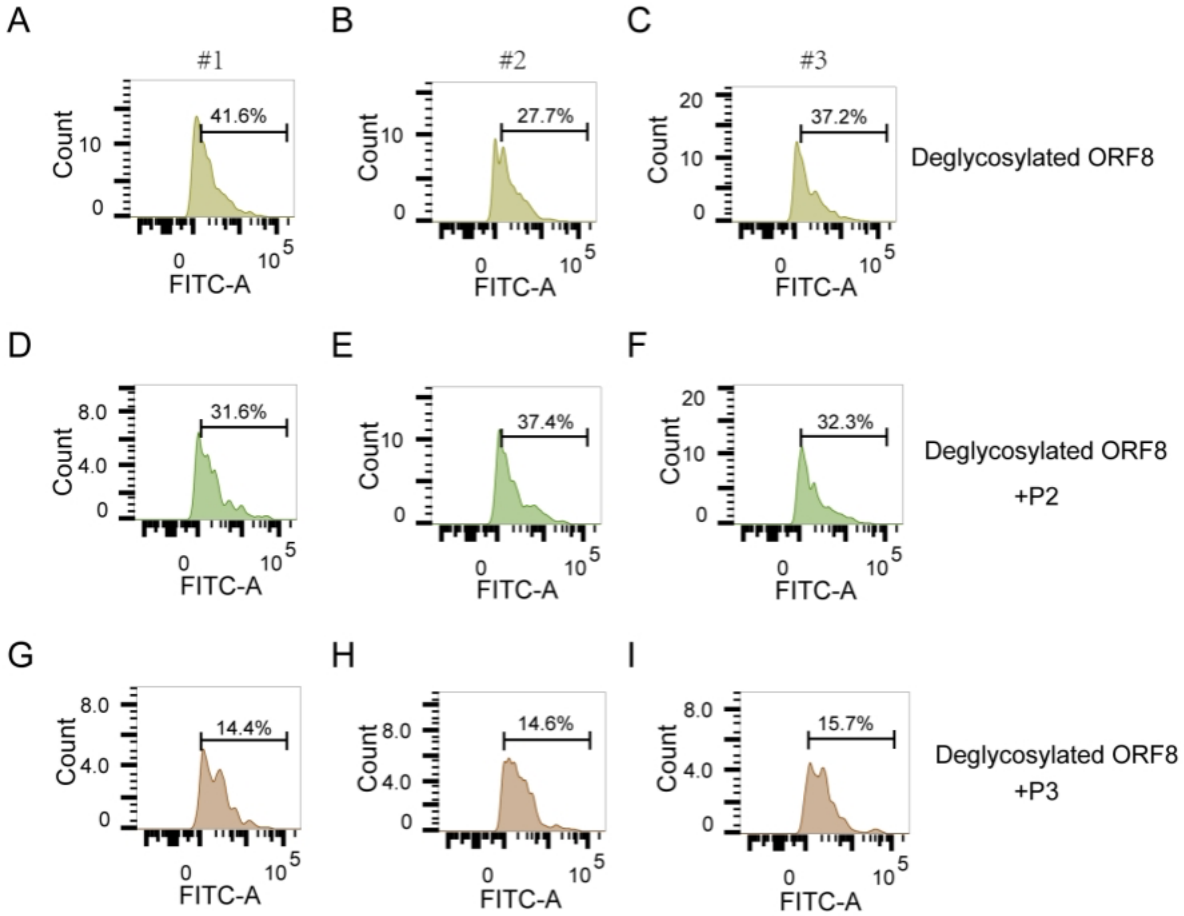

Figure S3 Flow cytometric histogram of ORF8 binding to immune cells after peptides P1 and P2 inhibition of deglycosylation. (**A-C**) The histogram of ORF8 binding to immune cells after deglycosylation is shown. (**D-F**) The histogram of the inhibitory effect of peptide P2 on the binding of deglycosylated ORF8 to immune cells is presented. (**G-I**) The histogram of the inhibitory effect of peptide P3 on the binding of deglycosylated ORF8 to immune cells is presented. The figure displays three replicate experiments.

Table S1. Information about the peptides involved in the manuscript.

Name	Source	Length	Sequence
P1	ADAM9	12 (aa580-591)	EIPVFGIVPAII
P2	ADAMTS1	10 (aa781-789)	GDYTLSTLEQ
P3	IL17RA	10 (aa89-98)	TNERLCVRFE
P4	MHC-I	16 (aa86-101)	GETRKVKAHSQTHRVD
P5	MHC-I	18 (aa170-187)	KWEAAHVAEQLRAYLEGT

Table S2. Amino acid mutations of C20 for ORF8 stabilization.

Amino acid	Predicted ΔΔG (kcal/mol)	Overall Stability
C20G	-4.61	Destabilizing
C20A	-7.2	Destabilizing
C20V	-7.39	Destabilizing
C20L	-5.57	Destabilizing
C20I	-4.81	Destabilizing
C20M	-1.99	Destabilizing
C20P	-4.27	Destabilizing
C20W	-0.56	Destabilizing
C20S	-6.73	Destabilizing
C20T	-7.34	Destabilizing
C20F	-3.85	Destabilizing
C20Q	-4.87	Destabilizing
C20K	-4.28	Destabilizing
C20Y	-3.77	Destabilizing
C20N	-2.78	Destabilizing
C20E	-3.7	Destabilizing
C20D	-5.53	Destabilizing
C20R	-1.01	Destabilizing
C20H	-3.85	Destabilizing

Table S3. Amino acid mutations of C25 for ORF8 stabilization.

Amino acid	Predicted ΔΔG (kcal/mol)	Overall Stability
C20G	-5.89	Destabilizing
C20A	-5.2	Destabilizing
C20V	-7.55	Destabilizing
C20L	-7.3	Destabilizing
C20I	-8.59	Destabilizing
C20M	-5.55	Destabilizing
C20P	-13.53	Destabilizing
C20W	-4.16	Destabilizing
C20S	-5.17	Destabilizing
C20T	-6.27	Destabilizing
C20F	-6.56	Destabilizing
C20Q	-6.55	Destabilizing
C20K	-9.83	Destabilizing
C20Y	-6.38	Destabilizing
C20N	-7.07	Destabilizing
C20E	-6.82	Destabilizing
C20D	-7.71	Destabilizing
C20R	-3.39	Destabilizing
C20H	-6.51	Destabilizing

Table S4. Amino acid mutations of C37 for ORF8 stabilization.

Amino acid	Predicted ΔΔG (kcal/mol)	Overall Stability
C20G	-4.95	Destabilizing
C20A	-6.87	Destabilizing
C20V	-6.25	Destabilizing
C20L	-10.45	Destabilizing
C20I	-6.13	Destabilizing
C20M	-9.38	Destabilizing
C20P	-0.63	Destabilizing
C20W	-2.18	Destabilizing
C20S	-4.85	Destabilizing
C20T	-6.5	Destabilizing
C20F	-8.88	Destabilizing
C20Q	-1.66	Destabilizing
C20K	2.67	Stabilizing
C20Y	-10.02	Destabilizing
C20N	-1.74	Destabilizing
C20E	-4.44	Destabilizing
C20D	-4.57	Destabilizing
C20R	-3.21	Destabilizing
C20H	1.33	Stabilizing

Table S5. Amino acid mutations of C61 for ORF8 stabilization.

Amino acid	Predicted ΔΔG (kcal/mol)	Overall Stability
C20G	-4.34	Destabilizing
C20A	-6.89	Destabilizing
C20V	-5.68	Destabilizing
C20L	-6.78	Destabilizing
C20I	-6.36	Destabilizing
C20M	-5.44	Destabilizing
C20P	-7.29	Destabilizing
C20W	-5.24	Destabilizing
C20S	-5.5	Destabilizing
C20T	-7.14	Destabilizing
C20F	-5.87	Destabilizing
C20Q	-5.37	Destabilizing
C20K	-5.84	Destabilizing
C20Y	-5.15	Destabilizing
C20N	-3.38	Destabilizing
C20E	-3.81	Destabilizing
C20D	-3.65	Destabilizing
C20R	-3.84	Destabilizing
C20H	-1.56	Destabilizing

Table S6. Amino acid mutations of C83 for ORF8 stabilization.

Amino acid	Predicted ΔΔG (kcal/mol)	Overall Stability
C20G	0.69	Stabilizing
C20A	-0.26	Destabilizing
C20V	-1.49	Destabilizing
C20L	-0.75	Destabilizing
C20I	-2.43	Destabilizing
C20M	-0.8	Destabilizing
C20P	-2.09	Destabilizing
C20W	0.56	Stabilizing
C20S	-0.53	Destabilizing
C20T	-1.07	Destabilizing
C20F	0.27	Stabilizing
C20Q	0.04	Stabilizing
C20K	-1.36	Destabilizing
C20Y	-0.48	Destabilizing
C20N	-0.38	Destabilizing
C20E	0.16	Stabilizing
C20D	-0.82	Destabilizing
C20R	-0.89	Destabilizing
C20H	0.56	Stabilizing

Table S7. Amino acid mutations of C90 for ORF8 stabilization.

Amino acid	Predicted ΔΔG (kcal/mol)	Overall Stability
C20G	2.33	Stabilizing
C20A	1.91	Stabilizing
C20V	-2.49	Destabilizing
C20L	-1.31	Destabilizing
C20I	-2.91	Destabilizing
C20M	0.55	Stabilizing
C20P	2.16	Stabilizing
C20W	-1.73	Destabilizing
C20S	0.4	Stabilizing
C20T	-0.87	Destabilizing
C20F	2.13	Stabilizing
C20Q	0.69	Stabilizing
C20K	5.19	Stabilizing
C20Y	2.13	Stabilizing
C20N	0.4	Stabilizing
C20E	-0.5	Destabilizing
C20D	-0.55	Destabilizing
C20R	3.44	Stabilizing
C20H	2.97	Stabilizing

Table S8. Amino acid mutations of C102 for ORF8 stabilization.

Amino acid	Predicted ΔΔG (kcal/mol)	Overall Stability
C20G	-5.96	Destabilizing
C20A	-4.15	Destabilizing
C20V	-4.08	Destabilizing
C20L	-4.29	Destabilizing
C20I	-3.89	Destabilizing
C20M	-3.38	Destabilizing
C20P	-7.95	Destabilizing
C20W	-5.11	Destabilizing
C20S	-6.27	Destabilizing
C20T	-4.96	Destabilizing
C20F	-5.13	Destabilizing
C20Q	-5.48	Destabilizing
C20K	-4.95	Destabilizing
C20Y	-4.93	Destabilizing
C20N	-6.56	Destabilizing
C20E	-5.63	Destabilizing
C20D	-7.17	Destabilizing
C20R	-5.88	Destabilizing
C20H	-9.02	Destabilizing

Table S9. Alphafold2 predict the interaction between ORF8 and IL-17RA.

Source of ORF8	Predicted Binding to IL-17RA	Score (ipTM+pTM)
SARS-CoV-2 WH-Human1	Yes	0.4073
Bat CoV RaTG13	Yes	0.2455
SARS-CoV A22	Yes	0.4498
SARS-CoV Urbani	Yes	0.3595
SARS-CoV Tor2	Yes	0.3193
MERS-related CoV NL13845	Yes	0.1988
HCoV-HKU1 N2	Yes	0.2379
